# Vertebral Fractures among Patients Referred for Bone Densitometry Screening in Dubai Primary Health Care Facilities

**DOI:** 10.1155/2019/7974534

**Published:** 2019-03-06

**Authors:** Anood Jamal Alshaali, Soha Abd El Aziz Abd El Aal, Amal Mohamad AlJaziri, Tamer Mohamed Farid Abdellatif, Manal Mohammad Omran Taryam, Nahed AbdulKhaleq Monsef

**Affiliations:** ^1^Elderly Care Unit, Health Affairs Department, Primary Health Care Services Sector, Dubai Health Authority, Dubai, UAE; ^2^Primary Health Care Services Sector, Dubai Health Authority, Dubai, UAE; ^3^Strategy & Governance Department, Dubai Health Authority, Dubai, UAE

## Abstract

Vertebral fractures are one of the most common fractures associated with low bone mineral density. However two-thirds to three-fourths of patients with vertebral fractures are not clinically recognized. The objective of this study was to determine the prevalence of vertebral fractures in patients referred for bone densitometry and the most common site of fracture. The study was carried out in the osteoporosis clinic in Dubai primary health care center. A total of 120 patients were examined using the dual energy X-ray absorptiometry. Of all the patients, 48.3% were osteoporotic and 40.9% were osteopenic. The overall prevalence of vertebral fracture was 14.2%. The result showed that the prevalence of vertebral fracture was higher in female compared to male (15.7% and 9.7%, respectively). It was found that patients aged 80 and above had the highest prevalence of vertebral fracture (54.5%). Undiagnosed vertebral fractures were common. Therefore, it is crucial to prevent vertebral fracture through early diagnosis and appropriate treatment of osteoporosis.

## 1. Introduction

Osteoporosis is defined as a skeletal disorder characterized by compromised bone strength, predisposing a person to an increased risk of fracture. Osteoporosis and osteoporotic fractures are a major health care problem [1]. Worldwide, one in three women and one in five men over the age of 50 will suffer at least one osteoporotic fracture in their lifetime as reported by the international osteoporosis foundation [2]. In the USA, it is estimated that 2 million osteoporosis-related fractures occurred in 2005 and expected to exceed 3 million by 2025 [3].

Forearm, vertebral, and hip fractures are the most frequent osteoporotic fractures [4]. Acute vertebral fractures occur when the weight of the upper body exceeds the ability of the bone within the vertebral body to support the load. Vertebral fractures are common; however two-thirds to three-fourths of patients with vertebral fractures are not clinically recognized [5, 6]. It appears that only patients with clinical problems of vertebral fractures including severe and chronic back pain, height loss, spinal deformity, and disability come to clinical attention [7]. A study in Sweden reported that approximately 23% of vertebral deformities come to clinical attention in women [8].

The vertebral fracture status is a powerful and independent risk factor for all new fractures. Having one or more vertebral fractures leads to a fivefold increase in the patients risk of developing another vertebral fracture. These fractures increase mortality and morbidity and decrease quality of life [9].

The magnitude of the problem in United Arab Emirates has not been fully assessed. There is a paucity of published data on vertebral fractures in Arab countries in general and in the Gulf Area in particular. A study conducted among postmenopausal women in Saudi Arabia reported that the prevalence of vertebral fracture in women over the age of 50 years was 20.3% [10]. A second study conducted among male Saudi Arab in the eastern province showed that the prevalence of vertebral fractures was 13.1% [11].

Since the vertebral fracture is a significant risk factor for further fracture and the majority of patients with vertebral fracture remain undetected, this study is conducted to determine the prevalence of non-clinically recognized vertebral fracture in patients referred for bone densitometry and the most common site of fracture.

## 2. Methodology

The study was conducted in osteoporosis clinic in the primary health care center in Dubai Health Authority during 5-month study period (April–September 2015); all newly postmenopausal women and men ≥ 50 years referred for bone densitometry assessment for osteoporosis screening were included in the study.

### 2.1. Online Risk Assessment Osteoporosis Form

The Dubai Health Authority has an electronic medical system. The osteoporosis form is the tool used by family physician in the primary health care centers to screen postmenopausal women and men aged ≥ 50 years for osteoporosis. The form aimed to elicit the following information. Age, sex, postmenopausal history for female and hypogonadism history for male, history of chronic diseases related to osteoporosis (malignancies, chronic lung diseases, rheumatoid arthritis, liver disease, diabetes mellitus, lupus, malabsorption, inflammatory bowel, and eating disorders), organ or bone marrow transplant, chemotherapy, prolonged loss of mobility, fracture with minor trauma, glucocorticoid therapy ≥ 3 months, and radiological finding of osteopenia/ osteoporosis.

#### 2.1.1. Low Risk

Males < 70 years and females < 65 years with no positive risk factor on osteoporosis screening form were considered to have low risk.

#### 2.1.2. High Risk

 The following were considered to have high risk:

males ≥ 70 years and females ≥ 65 years;

postmenopausal women with one of the following risks:

- not using HRT,
- family history of traumatic fractures in first degree relatives,
- BMI ≤ 20,
- surgical or natural menopause before age of 40;

those with chronic diseases related to osteoporosis;

men with hypogonadism more than 5 years;

those with prolonged severe loss of mobility (unable to ambulate outside of one's dwelling without a wheelchair for greater than one year);

those receiving chemotherapy;

those with organ or bone marrow transplant.

#### 2.1.3. Very High Risk

 The following were considered to have very high risk:

those having prior fracture with minor trauma (fall from standing height or less);

those who have been, or are anticipated to be, on glucocorticoid therapy for 3 or more months at a dose equivalent to or greater than 5 mg prednisone per day;

those with radiological findings of osteopenia/osteoporosis.

Those categorized as high risk and very high risk were referred to osteoporosis clinic for bone densitometry assessment and further assessment.

### 2.2. Bone Mineral Density (BMD) Measurement

The BMD of the hip and the lumbar spine was measured using dual energy X-ray absorptiometry (DEXA) (Lunar, GE Health Care) and the result was expressed as T-scores. The reference standard of a T-score is the peak bone density, as reached in men or women between 20–30 years of age. The T-score is then defined as the number of standard deviations from this score. According to the WHO definition, “osteoporosis” is defined as a T-score equal to or lower than −2.5, “osteopenia” is defined as a T-score between −2.5 and −1.0, and when the T-score is equal to or greater than −1.0 BMD is “normal” [12].

### 2.3. Vertebral Fracture Assessment (VFA)

Immediately after BMD measurements VFA was performed. The new developments in the DEXA device allow assessment of vertebral fracture status using the same machine used for the BMD measurement. Vertebral fracture assessment by DEXA provides an image of the thoracic and lumbar spine for detecting vertebral fracture deformities [13].

### 2.4. Data Analysis

Statistical Package for social science (SPSS) program version 20 was used for analysis of data as follows:

- Descriptive statistics were carried out in the form of mean, standard deviation, and range for quantitative values.
- Frequency and percentage were done for qualitative variables

## 3. Results

The present study comprised 120 patients; the highest percentage of patients were in the age range 60 to 69 years (29.2%) followed by those aged 70-79 years (25.8%). Approximately 75 % of patients were female and the majority were UAE national (90.0%) (Table 1).

Table 2 shows that almost half of the patients (48.3%) who were newly referred to the osteoporosis clinic were osteoporotic. Furthermore, 17 out of 120 of the patients had no clinically recognized vertebral fractures.

The prevalence of vertebral fractures was higher among patients aged 80 and above (54.5%) than those aged 70-79 or 60-69 years (25.8% and 5.7%, respectively) as revealed in Table 3. It was found that 29.3% of patients with osteoporosis had vertebral fracture.

**Figure 1:**
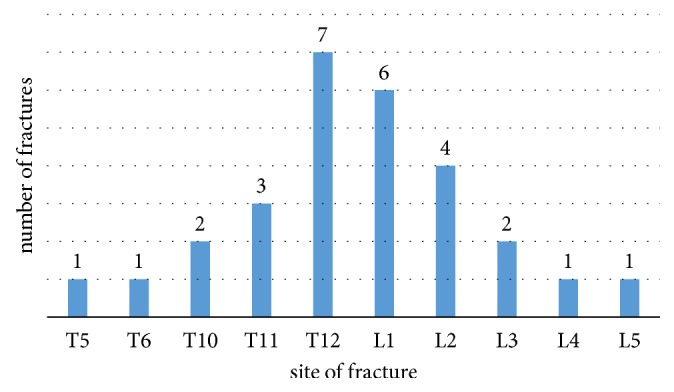
Distribution of vertebral fracture (VF) within the spine for newly referred patients to the osteoporosis clinic.

Figure 1 shows the distribution of vertebral fracture within the spine for newly referred patients to the osteoporosis clinic. Fractures were seen from the 5^th^ thoracic to the 5^th^ lumbar vertebrae, most common at the 12^th^ thoracic vertebrae and the 1^st^ lumbar vertebrae. There were seven fractures in the 12^th^ thoracic vertebrae and six fractures in the 1^st^ lumbar vertebrae.

**Figure 2:**
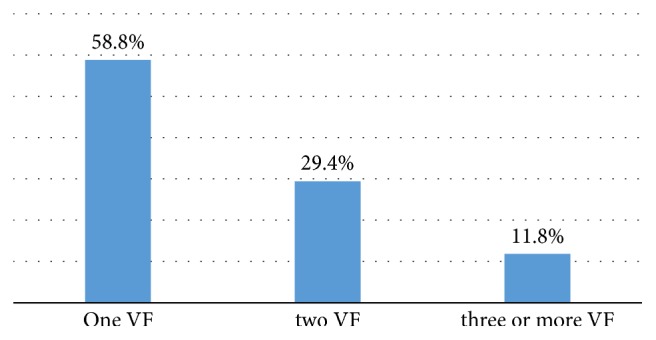
Distribution of number of fractures in patients assessed with vertebral fracture.

Figure 2 shows the distribution of number of fractures in patients assessed with vertebral fracture. More than half (58.8%) had one vertebral fracture and almost one-third (29.4) had two vertebral fractures. The data showed that 10 patients had one vertebral fracture, 5 patients had two vertebral fractures, and 2 patients had three or more vertebral fracture.

## 4. Discussion

Osteoporosis and osteoporotic fractures are a major public health problem all over the world. The probability of sustaining osteoporotic fractures varies markedly in different regions of the world. The burden of the fracture may become larger in the Middle East region where the prevalence of low bone mass is higher than in western countries [14].

Screening for osteoporosis in primary health care centers in Dubai is done by family physician for postmenopausal women and men at age 50 years and above using the online risk assessment osteoporosis form. Patients identified as having high risk were referred to osteoporosis clinic for dual energy X-ray absorptiometry (DEXA) and further assessment.

In this study, the overall prevalence of vertebral fracture was 14.2, which is in general agreement with other studies in the region showing an overall prevalence ranging from 12.6% to 17.1% [15–17]. But this prevalence is considered to be high if compared to that of western countries (14).

Vertebral compression fractures affect many patients worldwide and are most common in elderly population [5]. In the present study, the prevalence of vertebral fracture increased as patients aged. This finding is consistent with previous studies in UK [18] and Norway [15] where older individuals had a higher prevalence of vertebral fracture than younger individuals. With the new phenomenon of a “graying population” and the aging of the United Arab Emirates population, the number of fractures is expected to rise unless appropriate screening measures are implemented and adequate treatment is given.

Regarding sex distribution, previous studies in Netherland [19], China [20], and Lebanon [17] showed that vertebral fracture was more common in women. These findings are in accordance with our result, which revealed that the prevalence of vertebral fracture was higher in women in comparison with men (15.7% and 9.7%, respectively). The difference in men and women may be caused by the fact that overall men have a higher peak BMD and loose bone at a lower rate than women do [21].

This study suggests that osteoporosis is associated with vertebral fracture [22]. This is illustrated by our finding that all patients with vertebral fracture were found to be osteoporotic. Furthermore, in our study, the skeletal location of vertebral fracture is highest in T 12 and L1; this is in agreement with previous studies carried out in Netherland [13, 19].

### 4.1. Strength

The prevalence of vertebral fracture was identified.

The vertebral fracture assessment was done using the new developments in the DEXA device.

### 4.2. Limitation

The sample size was small. It would be more convincing if a study with a larger sample size was conducted. Despite the obvious limitation, this study provides useful data for prevention planning.

## 5. Conclusion

Undiagnosed vertebral fractures are common. Approximately one in seven patients referred for bone densitometry screening had a vertebral fracture. The prevalence increased as patients aged reaching 54.5% for those aged 80 and above. These fractures increase mortality and morbidity and decrease quality of life. Therefore, it is crucial to prevent vertebral fracture through early diagnosis and appropriate treatment of osteoporosis.

## Figures and Tables

**Table 1:** Distribution of patients attending the osteoporosis clinic according to personal characteristics.

Personal characteristics	n=120	(%)
Age		
<50	14	11.7
50-59	29	24.2
60-69	35	29.2
70-79	31	25.8
≥80	11	9.2
Mean ± SD	63.48±12.59	
Range	45-93	

Sex		
male	31	25.8
female	89	74.2

Nationality		
UAE national	108	90.0
non- UAE national	12	10.0

Body Mass Index (BMI)		
Underweight <18.5	1	0.8
Normal 18.5 -24.99	32	26.7
Overweight ≥25	45	37.5
Obese ≥30	42	35.0
Mean ± SD	28.69±5.73	
Range	17.5-40	

**Table 2:** Bone Mineral Density (BMD) classification and the prevalence of vertebral fractures.

DEXA result	n=120	%
BMD classification		
Normal	13	10.8
Osteopenia	49	40.9
Osteoporosis	58	48.3

Vertebral fracture		
No	103	85.8
Yes	17	14.2

**Table 3:** Prevalence of vertebral fractures according to age, sex, and BMD.

Vertebral fractures	n= 17	%
Age		
<50	0	0
50-59	1	3.4
60-69	2	5.7
70-79	8	25.8
≥80	6	54.5

Sex		
female	14	15.7
male	3	9.7

BMD		
Normal	0	0
Osteopenia	0	0
Osteoporosis	17	29.3

## Data Availability

The data used to support the findings of this study are available from the corresponding author upon request.
